# MMBGR protocol – diagnostic accuracy of clinical examination in preschoolers

**DOI:** 10.1590/2317-1782/e20250141en

**Published:** 2026-08-03

**Authors:** Anna Luiza dos Santos Matos, Giédre Berretin-Felix, Katia Flores Genaro, Íkaro Daniel de Carvalho Barreto, Ricardo Queiroz Gurgel, Andréa Monteiro Correia Medeiros

**Affiliations:** 1 Programa de Pós-graduação em Ciências da Saúde, Universidade Federal de Sergipe – UFS - Aracaju (SE), Brasil.; 2 Faculdade de Odontologia de Bauru, Universidade de São Paulo – USP - Bauru (SP), Brasil.; 3 Centro Brasileiro de Pesquisa em Avaliação e Seleção e de Promoção de Eventos – CEBRASPE - Brasília (DF), Brasil.; 4 Departamento de Fonoaudiologia, Universidade Federal de Sergipe – UFS - São Cristóvão (SE), Brasil.; 5 Programa de Pós-graduação em Saúde da Comunicação Humana - PPGSCH, Universidade Federal de Pernambuco - UFPE - Recife (PE), Brasil

**Keywords:** Speech, Language and Hearing Sciences, Preschoolers, Clinical Diagnosis, Validation Study, Sensitivity and Specificity, Myofunctional Therapy, Stomatognathic System

## Abstract

**Purpose:**

To present the diagnostic accuracy of the scored Clinical Examination of the MMBGR Orofacial Myofunctional Assessment Protocol for the age range of 24 to 71 months.

**Methods:**

Diagnostic accuracy validation study with a convenience sample. Fourteen speech-language pathologists analyzed images of 132 participants from the database of previous validation stages, divided into two groups (G1 = 24 to 35 months and 29 days; G2 = 36 to 71 months and 29 days). Each image was analyzed individually and independently by a panel of three expert speech-language pathologists, with agreement between at least two evaluators considered valid. Opinions regarding domains of the orofacial myofunctional examination, Orofacial Myofunctional Disorder (OMD), and referral needs were recorded using an electronic form. The speech-language pathologists’ responses based on clinical experience, without using the protocol (gold standard), were compared with those obtained using the Protocol (index test). Receiver Operating Characteristic (ROC) curve analysis was applied, cutoff points were established, and sensitivity and specificity values were obtained using R Core Team software (2022).

**Results:**

Diagnostic accuracy in preschool children in G1 was ideal and in G2 was reasonable for orofacial structures. For orofacial functions, accuracy was ideal in G1 and reasonable in G2; for tone, accuracy was reasonable in both groups. For OMD, accuracy was reasonable for both G1 and G2. The cutoff point for OMD was 15 (24–35 months) and 22 (36–71 months). Accuracy was reasonable for predicting multidisciplinary and speech-language pathology referrals.

**Conclusion:**

The MMBGR Protocol demonstrated adequate diagnostic accuracy for orofacial structures and functions in preschool children. It showed reasonable accuracy for diagnosing OMD and for predicting multidisciplinary and speech-language pathology referrals.

## INTRODUCTION

Speech-Language Pathology is a science undergoing extensive development, with clinical practice playing a central role in the pursuit of diagnostic instruments for diverse populations. In the field of Orofacial Myofunctional Therapy (OMT), the proposition and growing interest in validated protocols targeting younger age groups have been observed, particularly over the past five years, as exemplified by the AMIOFE-E for infants^(1)^ and the MMBGR Protocol for infants and preschoolers^(2,3).^

Clinical practice in OMT encompasses understanding the relationship between orofacial structures and functions during early childhood, given that this period is critical for early diagnosis and intervention. Knowledge of extraoral structures—including the lips, face, and mandible—and intraoral structures—such as the tongue, cheeks, hard palate, soft palate, palatine tonsils, and teeth—is required, in addition to tonus and functions of the stomatognathic system, so that the structural and functional interrelationships may be determined^(4,5)^.

The structures, encompassing musculature, soft tissues, bones, and teeth, and their relationships with orofacial functions, play a significant role in orofacial growth and development through adulthood. Facial motricity has phonetic, deglutitory, respiratory, and mimetic repercussions, and its equilibrium results in functional harmony^(6)^. There is a need to expand standardized instruments aimed at the orofacial myofunctional assessment of the pediatric population, which have been validated as recommended for tests in Speech-Language Pathology^(7).^

Standardized protocols must consistently evaluate what they propose; therefore, undertaking validation steps—such as diagnostic accuracy—is fundamental for establishing diagnostic measures and indicating the presence or absence of disorders, reflecting on normality and including score assignment. During the accuracy stage, results from the intended test (index test) must be compared against the test considered the reference standard (gold standard). This comparison should indicate the predictive capacity of the index test in anticipating results, according to sensitivity, specificity, and predictive values^(8,9)^. In Speech-Language Pathology, to date, no other validated protocol for the preschool population existed that could serve as a comparative instrument (gold standard) for research purposes, making it necessary to employ a reference-standard alternative.

According to STARD, the reference standard is defined as "the best method available for establishing the presence or absence of the target condition" (item 10–11). Thus, in the absence of already standardized and validated instruments, and populations without an established diagnosis, the determination of a "*clinical reference standard*" may be undertaken through the interpretative judgment of clinicians who attend the age group, with recognized expertise in the field^(10)^.

Currently, the MMBGR Protocol^(2,3)^ is available for assessing orofacial myofunctional aspects in childhood, with some validation phases already completed. However, until the present moment, the diagnostic accuracy validation stage for preschoolers (24 to 71 months of age) had not been presented, with defining diagnostic measures that enable clinical decision-making, prognosis^(9)^ and referrals.

In this direction, the study presents the diagnostic accuracy validation stage for the preschool age range of the MMBGR Protocol: Orofacial Myofunctional Clinical Examination with scores and the protocol's assertiveness for speech-language pathology and/or multidisciplinary referrals.

## METHODS

A validation study of prospective design and convenience sampling, concerning the diagnostic accuracy of the Protocol^(3)^, targeting the age range of 24 to 71 months. Authorization was obtained from the authors of the original protocol, and the study was approved by the Research Ethics Committee (REC) of the Universidade Federal de Sergipe, under opinion number 5.147.320.

The diagnostic accuracy validation stage was made possible by the prior proposition of the instrument, occasion on which static and dynamic images were obtained from preschoolers attending public institutions (daycare centers and university hospitals) in two Brazilian states, one from the Southeast region and another from the Northeast region, through authorizations and signatures of the Informed Consent Forms (ICF). This image database had already been constituted by preschoolers who met the inclusion criteria of the previous study^(3)^, namely: absence of speech-language pathology complaints, neurological diagnoses, or developmental disorders, as indicated by the responsible parties of the data collection settings.

The sample size was determined with the objective of testing the hypothesis of diagnostic efficacy of the method under investigation. The statistical design sought to detect an expected sensitivity of 80% (H1), in contrast to a null hypothesis of 50% (H0), maintaining a statistical power of 80% and a significance level of 5% (one-tailed). Uncertainty regarding the true proportion of affected individuals in the accessed population was considered. Assuming an estimated prevalence of 50% for the clinical condition of interest, the application of the power and alpha error parameters into the sample size formula resulted in the definition of at least 36 participants to be included in the study. This quantity was established to provide adequate probability of rejection of the null hypothesis, should the diagnostic performance reach the expected magnitude^(11)^.

For the issuance of expert opinions in the present study, concerning the images of the preschoolers, speech-language pathologists were recruited based on the analysis of their Lattes curricula, according to the inclusion criteria of the study, which required the professional to be a specialist in OMT and to work with the preschool population. To this end, a survey of professionals listed as specialists on the website of the Federal Council of Speech-Language Pathology (CFFa) was conducted, across different regions of the country; subsequently, contact was established with each professional through instant messaging applications and electronic mail (e-mail). Those who expressed interest received, via e-mail, the materials containing the ICF and the link for its printing, as recommended by the REC.

Also by e-mail, a link was forwarded to the professional characterization form, where authorization for participation in the study was to be indicated, and another link corresponding to the completion of the assessment of the clinical cases from the database to be analyzed. Sharing of the cloud containing static and dynamic images of the preschoolers to be evaluated was performed.

The distribution of videos among the specialist speech-language pathologists was carried out randomly, respecting the distribution by age range; thus, the number of images varied according to the number of subjects belonging to each group (G1 or G2). The age range in which the professional worked was respected, maintaining the professional always with the same age group: G1 — 24 months to 35 months and 29 days (n=36); G2 — 36 months to 71 months and 29 days (n=96).

The professionals had autonomy to set their own pace of assessments. The researchers made themselves available for any eventual doubts, which could include difficulties encountered by the speech-language pathologists in performing the analyses within the estimated timeframe. Should this be the case, this deadline could be extended.

### Previous validation stages

The MMBGR Protocol underwent other validation stages, encompassing the age ranges of infants (six to 23 months) and preschoolers (24 to 71 months): content validity of the instrument and validity based on response processes with application of the Clinical Examination and test reliability^(2,3)^, during which the image database was constructed, according to the general guidelines contained in the MMBGR Protocol instructional manual for infants and preschoolers, following the protocol aspects to be considered according to the age range: with static (JPEG) and dynamic (MP4) recording^(2)^, using a digital camera (Panasonic Compact-VHS Palmcorder) hand-held, with close-up image of the orofacial region (Macro Led Ring Flash HD lens)^(3)^. All procedures described, including the recruitment of professionals, followed the same format as the research involving the study of diagnostic accuracy of the instrument for the infant age range^(10)^. That is, according to the data analysis and considering the results obtained, the presentation of diagnostic accuracy was chosen, in accordance with particularities related to the development of each age range, with the preschool population addressed herein.

### Constitution of participant groups (preschoolers) and assessed domains

The images (static and dynamic) of the preschoolers were distributed into two groups: G1 — 24 months to 35 months and 29 days (n=36); G2 — 36 months to 71 months and 29 days (n=96).

The group distribution followed the same standardization as the original protocol^(3)^, including regarding the items and domains assessed. Thus, all items analyzed were common to both age ranges, except for general aspects of phonetic-articulatory production (belonging to speech), for which the original instrument proposal is that they be considered from 36 months of age onwards. The instrument authors consider that there is a gradual maturation process of phonetic-articulatory production, such that under 36 months there are characteristics inherent to development that could not be evaluated and/or characterized as Orofacial Myofunctional Disorder (OMD), and that language aspects could also be further explored in specific protocols^(11-14)^.

Regarding the domains, the extraoral examination contained items related to the face, lips, and mandible; the intraoral examination, directed to the mucosa of the lips, cheeks, tongue, palate, palatine tonsils, teeth, and occlusion. There was also the issuance of expert opinion concerning data related to the tonus of the lips, tongue, chin region, and cheeks. For analyses of stomatognathic functions, dynamic images of the functions of breathing, mastication, liquid and solid deglutition, and speech were made available. As final items of the form, the evaluator should indicate, in the general domain, whether the assessed case/individual presented OMD or not, whether there was difficulty in completing the form, and whether there was need for speech-language pathology and/or multidisciplinary referrals (Dentistry, Otorhinolaryngology, among others).

### Diagnostic accuracy: index test and gold standard test

#### Index test

In the previous stage concerning validity based on response processes, with the execution of test reliability^(2,3)^, calibrated speech-language pathologists evaluated the images obtained from the preschoolers using the MMBGR Protocol, with inter- and intra-rater agreement^(3)^ of each instrument item being verified. The assessments (stored in the researcher's database) were considered in the present study as the "index test."

In the test referenced as the index, 100% of the sample was considered for examining inter-rater agreement, and intra-rater agreement approximately 20% to 30% of this sample, from each age range (39 cases randomly selected). These reassessments (retest) performed by the same rater occurred with a minimum interval of 15 days from the initial assessment, preventing the previous evaluation from influencing performance^(3)^. In this sense, inter- and intra-examiner agreement was utilized through the Intraclass Correlation Coefficient (ICC), according to the previous study, classified as poor (below 0.4), fair to good (between 0.4 and 0.7), and excellent (above 0.7)^(3)^.

In the analysis of inter-rater reliability, in the age range of 24–35 months, poor agreement was observed for the extraoral examination (ICC=0.26) and for the tonus item (ICC=0.13), whereas the intraoral examination presented excellent agreement (ICC=0.75); the respiration, mastication, deglutition, and speech items presented fair to good agreement, with ICCs of 0.40, 0.56, 0.62, and 0.44, respectively. In the age range of 36–71 months, the extraoral examination presented fair agreement (ICC=0.52), whereas the intraoral examination and the tonus item presented poor agreement (ICC=0.39 and 0.34, respectively); excellent agreement was observed for the respiration item (ICC=0.75) and maintenance of fair to good agreement for mastication, deglutition, and speech, with ICCs of 0.45, 0.49, and 0.65, respectively^(3)^.

In the analysis of intra-rater reliability, in the age range of 24–35 months, the extraoral examination, the intraoral examination, and the speech item presented excellent agreement (ICC=0.79; 0.88; 0.88, respectively), whereas the tonus (ICC=0.40), respiration (ICC=0.48), and deglutition (ICC=0.51) items presented fair to good agreement, and mastication showed poor agreement (ICC=0.38). In the age range of 36–71 months, excellent agreement was observed for the extraoral examination (ICC=0.87), tonus (ICC=0.75), respiration (ICC=1.00), deglutition (ICC=0.92), and speech (ICC=0.80) items, whereas the intraoral examination (ICC=0.65) and mastication (ICC=0.71) presented fair to good agreement^(3)^.

#### Gold standard test

The same images of preschoolers, stored in the researcher's database, were analyzed by a group of speech-language pathologists specialists in OMT, designated as the "committee of specialist speech-language pathologists (raters)," with clinical experience in the care of preschoolers and who could not have had prior contact with and/or used the MMBGR Protocol. The speech-language pathologists answered questions about their professional characterization and performed the analyses of the images, individually and separately, without the use of the MMBGR Protocol, these assessments being considered in the present study as the "gold standard test."

It was proposed that each case count on the assessment of a trio of specialists, who answered separately and individually two electronic forms. The first, "Professional Characterization," contained the questions: age range, region of practice, academic title, teaching activity, years of experience, and practice with preschoolers; the second referred to the "Orofacial Myofunctional Clinical Examination," and included the responses of the case assessments, based on the shared images; containing questions about the domains and eventual alterations. Regarding the specific domains: extraoral examination (face, lips, and mandible), intraoral examination (lips, cheeks, tongue, palate, palatine tonsils, teeth, and occlusion), and tonus (lips, tongue, chin region, and cheeks). For analyses of the orofacial functions: respiration, mastication, deglutition — liquid, solid, and semisolid — and phoneme production. As final items of the form, the rater should indicate whether the assessed case/individual presented OMT alterations, whether speech-language pathology treatment was needed, and whether referrals were necessary (Otorhinolaryngology and Dentistry). There was also the inquiry regarding the occurrence of problems in completing the form.

The responses obtained with the "Gold Standard Test" were tabulated and stored in a digital platform, analyzed intragroup, and agreement was expected regarding the presence or absence of OMD by at least two of the three specialists who analyzed each case. In the gold standard group, no scores were assigned in the assessment, with the rater merely stating dichotomously (yes/no) whether there was or was not alteration concerning the presented domain.

### Analyses for diagnostic accuracy validation

The employed methodology followed an approach similar to that used for the infant age range^(10)^, comparing the results obtained by the "gold standard test" and the "index test." The objective was to verify the effectiveness of the preschooler assessment process through the MMBGR Orofacial Myofunctional Assessment Protocol — Infants and Preschoolers (index test), when compared to the clinical assessment performed without the use of the aforementioned protocol (gold standard test), as well as to determine its predictive capacity.

For the validation of diagnostic accuracy, the determination of predictivity was essential. Thus, the Receiver Operating Characteristic (ROC) curve^(7)^ was employed for the identification of the area under the curve (AUC) and the definition of sensitive cutoff points for alterations in the items assessed by the MMBGR Protocol^(3)^.

From the identification of each cutoff point, sensitivity and specificity values were calculated. It is emphasized that there may be no relationship between OMD and the protocol scores by domain when the AUC equals 0.5, as evaluated by the Z test for proportions^(15)^. The AUC analysis allows estimating the probability of correct identification of individuals with alterations (true positives) and those without alterations (true negatives). AUC values close to 1.0 indicate better test performance, reflecting high sensitivity and specificity^(16)^.

Sensitivity represents the percentage of cases correctly identified as positive for alterations, being calculated by the sum of true positives and false negatives. Specificity, in turn, indicates the percentage of individuals correctly identified as non-carriers of the alteration, being calculated by the sum of true negatives and false positives^(17)^.

The positive predictive value (PPV) corresponds to the percentage of effectively altered individuals (true positives) calculated according to the sum of true positives and false positives. The negative predictive value (NPV) reflects the percentage of healthy individuals correctly identified by the test as non-carriers of alterations (true negatives), calculated according to the sum of true negatives and false negatives^(18,19)^.

The statistical analysis was conducted using R Core Team 2022 (Version 4.2.1). The results are presented objectively, with AUC representing the area under the ROC curve, 95% CI indicating 95% confidence interval, SE for sensitivity, SP for specificity, ACC for accuracy, in addition to TP (true positive), FN (false negative), FP (false positive), and TN (true negative). Furthermore, the Constrained Maximum Sensitivity technique was used, including the calculations of PPV and NPV.

For the interpretation of results, considering that a reduced volume of data may compromise confidence in classification, the values were adapted to the reality of the study. Thus, values below 60% were classified as "non-ideal," those between 60% and 80% as "fair," and those above 80% as "ideal"^(20)^.

## RESULTS

The results of the analyses performed by the specialist speech-language pathologists, who participated as raters, are presented below, distributed according to the analysis of the two age groups: G1 composed of 36 cases (age range of 24 to 35 months and 29 days) and G2 composed of 96 cases (age range of 36 to 71 months and 29 days).

The participation of 12 speech-language pathologists was estimated for the study, distributed across groups of preschoolers to be analyzed. However, it was necessary to expand the number of invitations, as during the course of the study there was a need for the entry of new raters to complement or replace those who, for extrinsic/personal reasons, declined participation before completing the analyses of all clinical cases. In total, 14 speech-language pathologists integrated the research.

On average, each specialist analyzed 31 to 40 cases, including static and dynamic images of the preschoolers that comprised the research database. Only the specialists who entered to complete the groups, due to the need for professional replacement, received the drive with a reduced number of cases, given that the research was already in progress.

During the study, the socioprofessional profile of the participants who composed the "committee of specialist speech-language pathologists (raters)" of the gold standard test was outlined, the data of which are presented in Table 1.

**Table 1:** Socioprofessional profile of the committee of specialist speech-language pathologists

**Age range**	**% (n)**
31 to 40 years	28.6 (4)
41 to 50 years	50.0 (7)
51 to 60 years	07.1 (1)
61 to 70 years	14.3 (2)
**Professional practice region**	
North	14.3 (2)
Northeast	28.6 (4)
Central-West	21.4 (3)
Southeast	28.6 (4)
South	07.1 (1)
**Academic title**	
Doctoral degree	50.0 (7)
Master's degree	35.7 (5)
Specialist	14.3 (2)
**Teaching activity (time of practice)**	
Non-teaching	21.4 (3)
Less than 5 years	07.1 (1)
Between 5 and 10 years	28.6 (4)
Between 11 and 15 years	14.3 (2)
Between 16 and 20 years	14.3 (2)
Between 21 and 25 years	00.0 (0)
More than 25 years	14.3 (2)
**Time of experience in SLP in OMT**	
Between 11 and 15 years	28.6 (4)
Between 16 and 20 years	14.3 (2)
Between 21 and 25 years	28.6 (4)
More than 25 years	28.6 (4)
**Time of practice in OMT with Preschoolers**	
Between 5 and 10 years	07.1 (1)
Between 11 and 15 years	35.8 (5)
Between 16 and 20 years	21.4 (3)
Between 21 and 25 years	07.1 (1)
More than 25 years	28.6 (4)

**Caption:** % = Percentage; n = number of observations

The following domains were established for the statistical analysis of OMT in preschoolers: orofacial structures (extraoral and intraoral), tonus, and orofacial functions (respiration, deglutition, mastication, and speech), with score assignment; in addition to domains regarding alterations indicative of OMD and the need for referral for speech-language pathology treatment and/or other multidisciplinary referrals.

**Figure 1:**
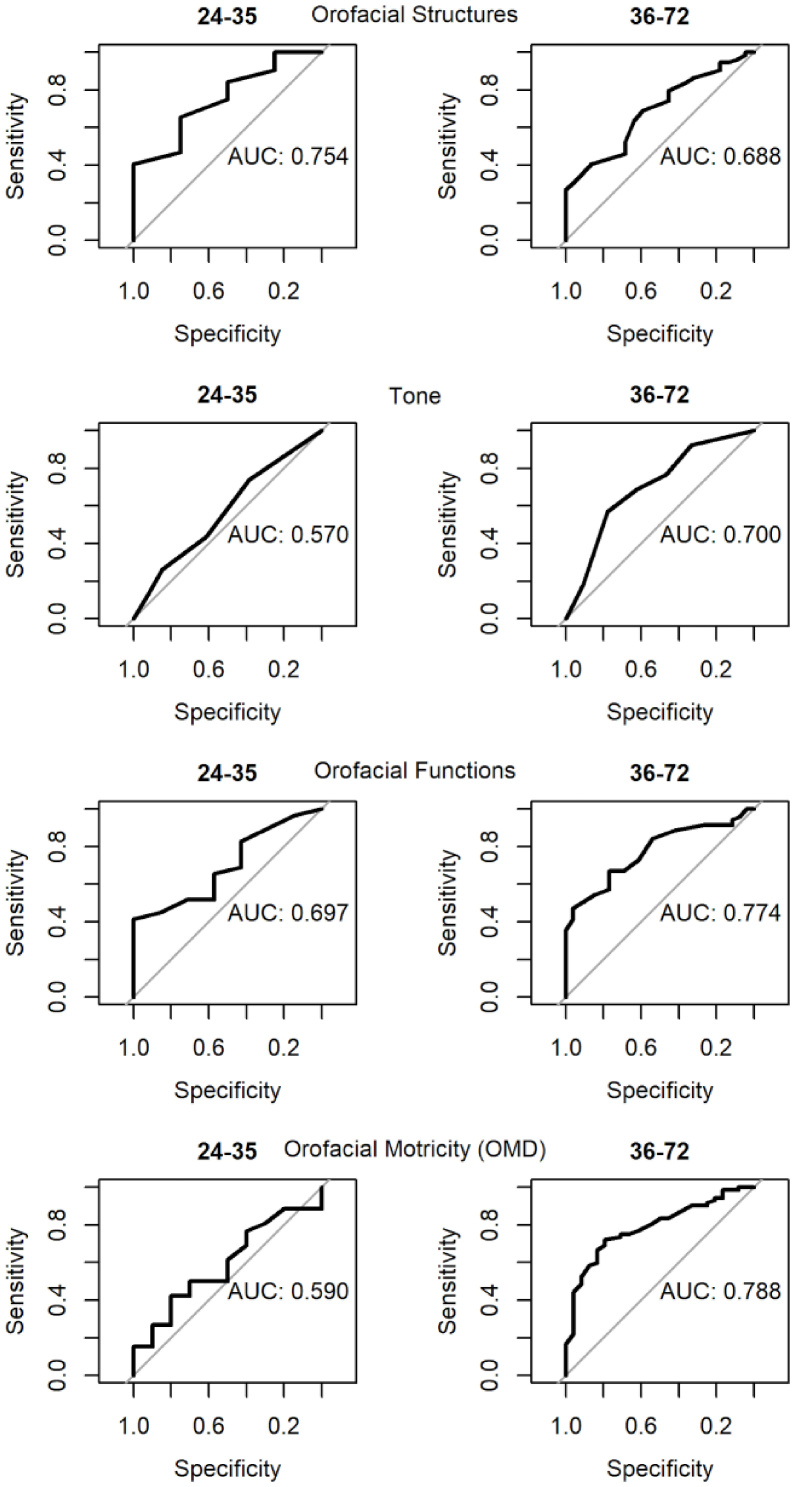
Graphical representation of the ROC curves of the domains of orofacial myofunctional assessment in preschoolers and referrals, according to age range (in months)

Regarding the domains pertaining to orofacial myofunctional assessment, Table 2 presents the values found from the analysis of the expert opinions issued by the committee of specialists, with corresponding graphical representation of the ROC curve in Figure 1.

**Table 2:** Diagnostic accuracy values, according to sensitivity and specificity, regarding domains of orofacial myofunctional assessment in preschoolers

**Domain**	**age Range (months)**	**AUC (95% CI)**	**Cutoff Value**	**Maximum Note**	**SE**	**SP**	**ACC**	**TP**	**FN**	**FP**	**TN**	**PPV**	**NPV**
Orofacial Structures	24-35	0.754 (0.499-1.000)	6	76	100	25.0	91.7	32	0	3	1	91.2	50.0
	36-71	0.688 (0.570-0.806)	8	76	96.0	9.1	76.4	71	3	20	2	78.0	40.0
Tonus	24-35	0.570 (0.376-0.764)	1	6	73.9	38.5	61.1	17	6	8	5	68.0	45.0
	36-71	0.700 (0.596-0.805)	1	6	92.2	33.3	64.6	47	4	30	15	61.0	78.9
Orofacial functions	24-35	0.697 (0.495-0.899)	7	53	96.5	14.3	80.6	28	1	6	1	82.3	50.0
	36-71	0.774 (0.679-0.869)	9	68	91.4	11.5	69.8	64	6	23	3	73.6	33.3
Orofacial Motricity (OMD)	24-35	0.590 (0.385-0.795)	15	135	96.1	0	69.4	25	1	10	0	71.4	0
	36-71	0.788 (0.691-0.886)	22	150	97.2	16.7	77.1	70	2	20	4	77.8	66.7

**Caption:** AUC = Area under the ROC curve; 95% CI = 95% Confidence Interval; SE = Sensitivity; SP = Specificity; ACC = Accuracy; TP = True Positive; FN = False Negative; FP = False Positive; TN = True Negative. Constrained Maximum Sensitivity. PPV = Positive Predictive Value; NPV = Negative Predictive Value; OMD = Orofacial Myofunctional Disorder

The summary of the Clinical Examination of the MMBGR Orofacial Myofunctional Assessment Protocol — Infants and Preschoolers, used for recording orofacial myofunctional assessment in preschoolers, with insertion of the respective cutoff points obtained in the present study, follows. (Chart 1)

**Chart 1:** Summary of the Orofacial Myofunctional Examination — MMBGR Infants and Preschoolers, with insertion of cutoff values by domains in preschoolers, according to age range

**Age range (months)**	**24-35** **(2 years)**	**36-71** **(3-5 years)**
**EXTRAORAL EXAMINATION (best result = 0 and worst = 20)**		
Face (*best result = 0 and worst = 10*)	[ ]	[ ]
Lips (*best result = 0 and worst = 9*)	[ ]	[ ]
Mandible (*best result = 0 and worst = 1*)	[ ]	[ ]
**INTRAORAL EXAMINATION (best result = 0 and worst = 56)**		
Lips (*best result = 0 and worst = 5*)	[ ]	[ ]
Cheeks (*best result = 0 and worst = 8*)	[ ]	[ ]
Tongue (*best result = 0 and worst = 16*)	[ ]	[ ]
Palate (*best result = 0 and worst = 10*)	[ ]	[ ]
Palatine tonsils (*best result = 0 and worst = 4*)	[ ]	[ ]
Teeth and Occlusion (*best result = 0 and worst = 13*)	[ ]	[ ]
**EXTRAORAL + INTRAORAL EXAMINATION RESULT**	**[ ]**	**[ ]**
**CUTOFF POINT: EXTRAORAL + INTRAORAL**	**6**	**8**
**TONUS (best result = 0 and worst = 6)**		
Lips (*upper + lower*) (*best result = 0 and worst = 2*)	[ ]	[ ]
Chin region (*best result = 0 and worst = 1*)	[ ]	[ ]
Tongue (*best result = 0 and worst = 1*)	[ ]	[ ]
Cheeks (*right + left*) (*best result = 0 and worst = 2*)	[ ]	[ ]
**TONUS RESULT**	**[ ]**	**[ ]**
**CUTOFF POINT: TONUS**	**1**	**1**
**OROFACIAL FUNCTIONS (best result = 0 and worst = 53/68)**		
Respiration (*best result = 0 and worst = 2*)	[ ]	[ ]
Sucking/Deglutition (*best result = 0 and worst = 22*)	___	___
Mastication (*best result = 0 and worst = 13*)	[ ]	[ ]
Semisolid/solid deglutition (*best result = 0 and worst = 17*)	[ ]	[ ]
Paste deglutition (*best result = 0 and worst = 22*)	___	___
Liquid deglutition (*best result = 0 and worst = 15*)	[ ]	[ ]
**OROFACIAL FUNCTIONS RESULT**	**[ ]**	**[ ]**
**CUTOFF POINT: OROFACIAL FUNCTIONS**	**7**	**9**
**TOTAL SCORE OBTAINED (Orofacial Myofunctional Clinical Examination)**	**[ ]**	**[ ]**
**CUTOFF POINT: Orofacial Myofunctional Disorder (OMD)**	**15**	**22**

Regarding the domains pertaining to referrals, Table 3 presents the values found from the analysis of the expert opinions issued by the committee of specialists.

**Table 3:** Diagnostic accuracy values, according to sensitivity and specificity, regarding referral domains for preschoolers

**Domain**	**Age Range** **(Months)**	**SE**	**SP**	**ACC**	**TP**	**FN**	**FP**	**TN**	**PPV**	**NPV**
Referral to Speech-Language Therapist	24-35	73.1	40.0	63.9	19	7	6	4	76.0	36.4
	36-71	77.3	63.3	72.9	51	15	11	19	82.2	55.9
Other multidisciplinary referrals	24-35	83.3	33.3	75.0	25	5	4	2	86.2	28.6
	36-71	84.3	23.1	76.0	70	13	10	3	87.5	18.7

**Caption:** SE = Sensitivity; SP = Specificity; ACC = Accuracy; TP = True Positive; FN = False Negative; FP = False Positive; TN = True Negative. Constrained Maximum Sensitivity. PPV = Positive Predictive Value; NPV = Negative Predictive Value

With the function of summarizing the diagnostic accuracy values and classification, according to domains of orofacial myofunctional assessment of preschoolers and referrals, Table 4 follows.

**Table 4:** Summary of diagnostic accuracy values and classification, according to domains of orofacial myofunctional assessment of preschoolers and referrals, by age range

**Domain**	**Accuracy/classification 24 to 35 months**	**Accuracy/classification 36 to 71 months**
Orofacial Structures	91.7 - Ideal	76.4 - Fair
Tonus	61.1 - Fair	64.6 - Fair
Orofacial Functions	80.6 - Ideal	69.8 - Fair
Orofacial Motricity (OMD)	69.4 - Fair	77.1 - Fair
Referral to Speech-Language Therapist	63.9 - Fair	72.9 - Fair
Other referrals (Multidisciplinary)	75.0 - Fair	76.0 - Fair

**Caption:** OMD = Orofacial Myofunctional Disorder

## DISCUSSION

The MMBGR Orofacial Myofunctional Assessment Protocol — Infants and Preschoolers is an important evaluative instrument in the field of OMT, with score assignment enabling the diagnosis of subjects with OMD for the age range of six to 71 months of life. According to the specificities involved, it was decided to present in the present manuscript data pertaining exclusively to the preschool population, including the detailing by subgroups according to age ranges (Group 1: 24 to 35 months and 29 days; Group 2: 36 to 71 months and 29 days).

The profile of the raters who composed the committee of specialists was exclusively female, with ages from 31 years onwards. The fact that they had experience in the field of OMT between 11 and more than 25 years, academic titles ranging from specialization in OMT to doctoral degrees, and experience reflecting broad expertise in the clinical care of preschoolers (the majority, 92.9%, with more than 11 years of practice with this age range) indicates that the images were analyzed by qualified professionals with a differentiated perspective for this population. The diversity of practice locations of the committee of specialists reached all regions of the federation, such that in the formation of trios for each age group, the raters were from different states, which allowed the analysis of the instrument to encompass distinct perspectives and experiences from the varied regions of Brazil.

It is considered that the professional profile of the committee of specialists, which encompassed vast teaching and clinical experience in the field of OMT with preschoolers, together with the diversity of practice locations, enabled the image analyses to be executed with the rigor prescribed for the procedures designed for validation, which increases the clinical value related to the instrument content and the level of scientificity of the work^(1)^.

The participation of 14 speech-language pathologists was necessary, due to the number of research participants; in order not to exceed the limit of images to be analyzed by each professional, a reasonable N was chosen, without causing exhaustion that would compromise the research and/or the wellbeing of the professionals.

Regarding the orofacial structures domain of the MMBGR Protocol, ideal results were obtained for sensitivity, being capable of identifying preschoolers with alterations in these structures, when altered. The specificity obtained was non-ideal, indicating that the test is poorly specific for predicting subjects without alteration in its absence. There was greater evidence in PPV than in NPV. This shows that the instrument is better able to identify preschoolers when there is alteration. It is considered that in preschoolers, alterations in orofacial structures are more evident than their absence; with the instrument being able to identify them assertively, fulfilling the role it proposes for diagnosis^(21)^.

The diagnostic accuracy for orofacial structures was considered ideal for the first age group and fair for preschoolers above 36 months, evidencing the capacity to detect the predicted subjects with alteration in both groups, with excellent exactitude in preschoolers of 24 to 35 months.

In tonus, sensitivity levels considered fair were obtained for preschoolers of 24 to 35 months and ideal for those of 36 to 71 months; with the instrument being sufficient in the detection of subjects with altered tonus, when there is in fact alteration. The non-ideal specificity for both age ranges indicates difficulty in identifying preschoolers without tonus alterations. In the older group, the PPV and NPV values evidenced that the instrument is able to identify in a fair manner both the presence and the absence of tonus alteration, which did not occur in the age group of 24 to 35 months, whose NPV was non-ideal, despite the PPV having been fair. On the other hand, given the good sensitivity values of orofacial structures, it is considered that their characteristics could assist in identifying tonus alterations, particularly in analyses performed through images. The absence of tonus alteration in younger subjects continues to be a challenge, when the examiner is deprived of direct palpation^(3,22)^.

The diagnostic accuracy in tonus remained fair for all age ranges, with the instrument having values considered sufficient in the exactitude of identifying what it proposed to assess. Even so, it is considered that the assessment obtained exclusively through video analysis may prejudice the diagnosis of tonus alterations. In the present research, the images were shared, and the raters instructed to analyze according to the criteria they used for the assessment of the other dynamic structures. Thus, the raters had freedom of interpretation according to their expertise; and even with the possible limitation, the rater performed the appraisal by viewing the dynamic image. New studies with probability sampling and use of the instrument to record the assessment in real time are recommended^(2,3)^.

The orofacial functions had sensitivity considered ideal for all age ranges, with the instrument proving effective in the identification of subjects with alteration in its presence. The specificity was non-ideal, with the instrument not being capable of identifying subjects without alterations in their absence. Once again, there was greater evidence in the proportion of PPV than NPV. The better identification of subjects with alteration, which did not occur in the unaltered cases, may be related to the physiological maturation of the orofacial functions themselves, such that the "atypical" patterns tend to be evident and have a greater chance of being diagnosed^(21)^. On the other hand, the real absence of alterations is not well detected by the instrument, which coincides with its objective of evaluating and diagnosing OMD, and not its absence.

The accuracy values of orofacial functions were fair for preschoolers of 36 to 71 months, and ideal for those of 24 to 35 months, evidencing that the instrument has value in diagnosis in both groups of preschoolers, with an important role in OMT clinical practice, reinforcing that the work with orofacial functions is within the expertise of the speech-language pathologist^(23)^.

The domain referring to OMT alteration concerns the presence or absence of OMD. Ideal sensitivity values and non-ideal specificity values were obtained for all age ranges of preschoolers. Thus, the instrument proved sufficient for identifying subjects with alterations and insufficient for ruling out those without alterations. The PPV indicated that all subjects with alterations were identified in a fair manner; however, there was discrepancy in the NPV values, when preschoolers of 36 to 71 months without alterations could be identified in a fair manner.

The findings regarding OMD are consistent with those evidenced in all other domains of the instrument, with the instrument having a greater capacity to predict subjects with alteration than its absence. Possibly, the development of the cranium and orofacial region, together with neurological, cognitive, and behavioral changes in the first years of life, influence the identification of existing problems, which tend to become more evident with advancing age^(22)^.

The accuracy result for OMD was fair for both age ranges, with the instrument being capable of diagnosing all preschoolers with alterations in their presence. This reinforces the importance of the validation performed regarding the Clinical Examination with scores, of the MMBGR Orofacial Myofunctional Assessment Protocol — Infants and Preschoolers, for the age range of 24 to 71 months, given that this material can assist the clinician in establishing an OMT diagnosis in a standardized and consistent manner.

The score values, referring to the cutoff points of each domain of the MMBGR Protocol, serve to indicate specific alterations by domain^(24)^, culminating in the final cutoff point, indicative of OMD.

Regarding orofacial structures, the instrument allows assigning scores from 0 (best result) to 20 (worst result) in the extraoral examination, and from 0 (best result) to 56 (worst result) for the intraoral; and the general cutoff score was established as 6 for G1 and 8 for G2. It is noted that low values, such as 6 and 8, already signal problems in the structures, which is important, considering the intrinsic relationship between alterations in structures and orofacial functions^(4)^ and their influence on the diagnosis of OMD.

Furthermore, this occurs for tonus, which establishes scoring between 0 (best result) and 6 (worst result), and the attainment of 1 point as the cutoff score already indicates alteration. It is notorious that alterations in tonus usually affect the functioning of the entire stomatognathic system^(4)^, which justifies the assignment of the low cutoff value in the protocol here studied^(23)^.

Considering the orofacial functions, the results range from 0 (best result) to 53 (worst result) for G1 and from 0 (best result) to 68 (worst result) for G2, with cutoff points of 7 (G1) and 9 (G2) having been established as indicative of alteration. Once again, low scores already signal to the clinician the need for attention, and make explicit the intimate relationship between structures and orofacial functions^(4)^.

Regarding the total scores for OMD, values of 15 (24 to 35 months and 29 days) and 22 (36 to 71 months and 29 days) were assigned to indicate the presence of the disorder and the need for speech-language pathology and/or multidisciplinary follow-up. Higher cutoff point values in older preschoolers coincide with the possibility of assigning higher scores in some specific domains of the protocol when ages are more advanced.

Having a protocol with scores, validated regarding its diagnostic accuracy, with establishment of a cutoff point for OMD is an advance in the field of OMT directed at the pediatric population, as it provides both diagnosis and enables envisioning the need for speech-language pathology and multidisciplinary referrals. The diagnosis and treatment for OMD at initial ages of life are relevant, as they can contribute to the full development of the human being in the other phases of life^(6)^.

In summary, the protocol focuses on the recording of aspects inherent to oromyofunctional assessment, according to the analysis of orofacial structures (static and mobile), tonus, and the respective orofacial functions, for the establishment of an OMD diagnosis, with score assignment. In addition, it also provides clinical reasoning regarding the need (or not) for referrals.

The capacity of the instrument to guide possible speech-language pathology and/or multidisciplinary referrals was analyzed based on its different domains, even though it did not involve the assignment of specific scores.

The need for referral to Speech-Language Pathology was assessed due to its relevance in the rehabilitation of individuals with possible alterations, contributing to the definition of adequate interventions. In both groups of preschoolers, the sensitivity was classified as fair, evidencing that individuals who needed referral were identified, and the specificity was considered non-ideal and fair, indicating that the identification of subjects without alteration is not so evident when these are younger, as occurred in the study performed with the infant age range^(10)^.

There was greater evidence in the proportion of PPV than in NPV, with the instrument being capable of guiding regarding the need for referrals in the presence of alterations. It is estimated that at more advanced ages, the possible orofacial myofunctional manifestations tend to be less tolerated by the clinician, resulting in a greater incidence of referrals^(4)^.

The diagnostic accuracy result in the indication of speech-language pathology follow-up for preschoolers was fair; thus, the instrument can be recognized as a reliable resource for determining the need for speech-language pathology referrals.

Regarding the need for multidisciplinary referrals (Otorhinolaryngology, Dentistry, among others), there was ideal sensitivity and non-ideal specificity, with the capacity of the instrument to identify preschoolers when there is in fact need for referral being more evident, in both age ranges. The PPV and NPV values reiterate the assertiveness in multidisciplinary referrals of subjects when there is presence of alteration. These data evidence that the protocol is capable of collaborating in the case direction to other professionals, which can assist in multidisciplinary diagnosis and enable the individual to be seen in a collaborative manner^(25,26)^.

The fair accuracy of the instrument for referrals to other professionals can be considered a positive aspect. Especially in the field of Dentistry, this accuracy may have had a relation with the nature of the items analyzed in the specific domains, with the prevalence of static images that facilitate analyses (teeth and occlusion and lingual frenulum), and in Otorhinolaryngology due to the ease of visualizing alterations in nasal airflow, through the use of the millimetered nasal mirror^(27,28)^. On the other hand, in the field of Otorhinolaryngology there is a probable difficulty of more detailed visualization of the palatine tonsils in the clinical examination by the speech-language pathologist, leading to the need for referral for more specific assessment.

The lack of referrals in the presence of alterations of the stomatognathic system may represent a risk to child development, particularly in cases whose approach demands diagnosis and intervention from the expertise of other health areas. In this sense, the capacity of the instrument to identify the need for multidisciplinary referral reinforces its value both for clinical practice in OMT in the specific speech-language pathology scope, and for interdisciplinary reasoning, promoting more comprehensive and integrated care.

In general, the findings evidence that the MMBGR Orofacial Myofunctional Assessment Protocol — Infants and Preschoolers is innovative for the preschool age range, as the diagnostic accuracy study allowed defining cutoff points, complementing the previously validated stages. It is highlighted that adequate sensitivity values were found in all domains of the instrument, demonstrating the capacity to identify alterations when present and to direct referrals, both speech-language pathology and multidisciplinary. The fact of presenting excellent sensitivity but low specificity for orofacial structures — a pattern that repeated in other domains (tonus, functions, OMD, referrals) may eventually indicate a tendency of the protocol to overestimate alterations (greater sensitivity than specificity), which must be interpreted with caution in clinical practice to avoid unnecessary referrals.

On the other hand, the results suggest that the use of the protocol can contribute to early detection and timely treatment of OMD, enabling better clinical outcomes and preventive strategies. It is considered that its use, as a standardized and validated instrument, should be encouraged so as to complement the evaluative process, together with the clinical examination, patient history, and other instruments, if necessary, strengthening evidence-based decision making and avoiding erroneous diagnoses.

The main difficulty of the study was regarding the recruitment of speech-language pathologists who were available to participate in the study, given the magnitude of work involved in the detailed analyses of the cases. In addition, the need to replace two speech-language pathologists for the conclusion of the cases delayed the research schedule, in relation to the predicted deadline for delivery of the analyses. These limitations were overcome with new invitations and authorizations, and redistribution of the drive of images of the cases not yet analyzed, enabling the conclusion of the diagnostic accuracy validation stage. However, in this context, there was the limitation of reassessment of the cases by the same rater, and it was not possible to perform intra-rater agreement, as occurred previously in the index test.

As a limitation of the study, the methodology of data analysis through recorded images (static and dynamic) does not always enable the best visualization and/or interpretation of a given assessed domain. It is recommended that speech-language pathologists, when using the MMBGR Protocol — Infants and Preschoolers in clinical routine, record the examination situation in real time, being able to check the obtained images subsequently, as recommended by the instrument authors^(2)^.

It is highlighted that the instrument demonstrated ideal sensitivity for the majority of domains in both age ranges. The only exception was the tonus domain in the age range of 24 to 35 months, classified as fair. This finding suggests the applicability of the instrument in real time, in addition to highlighting the importance of future development of complementary strategies for more precise assessment of this domain. The specificity, predominantly non-ideal, indicates that the instrument presents greater efficacy in the identification of subjects with alterations when these are present. In this context, it is highlighted that the objective of the MMBGR Protocol — Infants and Preschoolers is to detect individuals with alterations in a precise manner, suggesting it is a relevant tool both for clinical practice and for research in OMT.

The non-existence of a validated and graded reference standard for OMD is a recognized structural limitation in the field. According to the STARD (Standards for Reporting Diagnostic Accuracy Studies) recommendations, in situations in which there is no fully established "gold standard," it is adequate to employ the best clinical method available to determine the presence or absence of the condition. In the present study, a reference standard based on the consensus of three OMT specialists was used, a strategy described by STARD as a legitimate form of adjudication to reduce inter-observer variability. The dichotomous outcome adopted is consistent with the diagnostic question analyzed and with current clinical practice. Even so, it is recognized that the absence of validated severity instruments may influence accuracy estimates and reinforces the need for future development of more structured standards for the assessment of OMD^(29,30)^.

## CONCLUSION

The MMBGR Orofacial Myofunctional Assessment Protocol is an instrument for use in speech-language pathology clinical practice and research. The presented cutoff points enable the speech-language pathologist to identify OMD, utilizing the values as parameters for reassessments and follow-up of the preschooler.

The MMBGR Protocol presented overall fair accuracy for orofacial myofunctional assessment of preschoolers, with better performance for structures and functions in children of 24–35 months. The instrument also proved fair for supporting speech-language pathology and multidisciplinary referrals.

The results suggest potential utility in OMT clinical practice; however, given its non-ideal specificity regarding the identification of subjects without OMD, cautious clinical use is recommended, based on the expansion of multicenter studies in different populations. New cross-cultural validation studies of the protocol, including its scores, are pertinent.
