# Supplementary material for: MMBGR protocol – diagnostic accuracy of clinical examination in preschoolers
Source: Codas. 2026 Aug 3;38(5):e20250141. doi: 10.1590/2317-1782/e20250141en (PMC13445988; doi:10.1590/2317-1782/e20250141en)
Supplement: Translation

# Translation: Protocolo MMBGR – acurácia diagnóstica do exame clínico em pré-escolares

**DOI:** 10.1590/2317-1782/e20250141pt

**Authors:** Anna Luiza dos Santos Matos, Giédre Berretin-Felix, Katia Flores Genaro, Íkaro Daniel de Carvalho Barreto, Ricardo Queiroz Gurgel, Andréa Monteiro Correia Medeiros

**Affiliations:** 1 Programa de Pós-graduação em Ciências da Saúde, Universidade Federal de Sergipe – UFS - Aracaju (SE), Brasil.; 2 Faculdade de Odontologia de Bauru, Universidade de São Paulo – USP - Bauru (SP), Brasil.; 3 Centro Brasileiro de Pesquisa em Avaliação e Seleção e de Promoção de Eventos – CEBRASPE - Brasília (DF), Brasil.; 4 Departamento de Fonoaudiologia, Universidade Federal de Sergipe – UFS - São Cristóvão (SE), Brasil.; 5 Programa de Pós-graduação em Saúde da Comunicação Humana - PPGSCH, Universidade Federal de Pernambuco - UFPE - Recife (PE), Brasil

**Keywords:** Fonoaudiologia, Pré-escolar, Diagnóstico Clínico, Estudos de Validação, Sensibilidade e Especificidade, Terapia Miofuncional, Sistema Estomatognático

## Abstract

**Objetivo:**

Apresentar acurácia diagnóstica do Exame Clínico com escores, do Protocolo de Avaliação Miofuncional Orofacial MMBGR para faixa etária de 24 a 71 meses.

**Método:**

Estudo de validação da acurácia diagnóstica com amostra de conveniência. Quatorze fonoaudiólogos analisaram imagens de 132 participantes do banco de dados das etapas anteriores de validação, divididos em dois grupos (G1 = 24 a 35 meses e 29 dias; G2 = 36 a 71 meses e 29 dias). Cada imagem foi analisada por trio de fonoaudiólogos especialistas, individual e separadamente, sendo considerada válida concordância entre dois deles. Emitidos pareceres sobre domínios do exame miofuncional orofacial, Distúrbio Miofuncional Orofacial (DMO) e necessidade de encaminhamentos, em formulário eletrônico. Foram comparadas as respostas dos fonoaudiólogos baseadas na experiência clínica, sem utilizar o protocolo (padrão ouro) com aquelas emitidas com uso do Protocolo (teste índice). Utilizado método Receiver Operating Characteristic Curve (ROC), atribuídos pontos de corte e obtidos valores de sensibilidade e especificidade; software R Core Team 2022.

**Resultados:**

Acurácia diagnóstica em pré-escolares G1 é ideal e G2 razoável para estruturas orofaciais. Funções orofaciais acurácia ideal (G1) e razoável (G2), em tônus a acurácia foi razoável em ambos grupos. DMO a acurácia é razoável para G1 e G2.O ponto de corte para DMO é 15 (24 a 35 meses) e 22 (36 a 71 meses). A acurácia é razoável para encaminhamentos multidisciplinares e fonoaudiológicos.

**Conclusão:**

O Protocolo MMBGR apresentou acurácia adequada para estruturas e funções orofaciais de pré-escolares. É razoável para diagnosticar DMO e prever encaminhamentos multidisciplinares e fonoaudiológicos.

## INTRODUÇÃO

A Fonoaudiologia é uma ciência em amplo desenvolvimento, tendo a prática clínica como protagonista quanto à busca de instrumentos de diagnóstico em diferentes populações. Na área da Motricidade Orofacial (MO), têm-se observado, principalmente nos últimos cinco anos, a proposição e o interesse por protocolos que sejam validados e voltados para o público em idades mais precoces, como é o caso do AMIOFE-E lactentes^(1)^ e o Protocolo MMBGR – lactentes e pré-escolares^(2,3).^

A atuação na área de MO abrange compreender sobre a relação entre as estruturas e funções orofaciais ainda na infância, visto que é um período crucial para diagnóstico e tratamento de forma antecipada. Deve-se ter conhecimento das estruturas extraorais como lábios, face e mandíbula, e intraorais, como língua, bochechas, palato duro, palato mole e tonsilas palatinas, e dentes, além do tônus e funções do sistema estomatognático, para que a relação de estruturas e funções entre si possam ser determinadas^(4,5)^.

As estruturas, incluindo musculatura, tecidos moles, ósseos e dentes, e suas relações com as funções orofaciais, desempenham importante papel no crescimento e desenvolvimento orofacial até a fase adulta. A motricidade da face tem repercussões fonéticas, deglutitivas, respiratórias e mímicas, e seu equilíbrio resulta em harmonia funcional^(6)^. Existe a necessidade de ampliar os instrumentos padronizados, voltados para a avaliação miofuncional orofacial do público infantil, que tenham sido validados tal como preconizado para testes em Fonoaudiologia^(7).^

Protocolos padronizados precisam avaliar o que propuseram de modo consistente, por isso a realização de etapas de validação, tal como a acurácia diagnóstica, é fundamental para definir medidas de diagnóstico e sinalizar a presença ou não de distúrbios, refletindo sobre normalidade e incluindo a atribuição de escores. Na etapa de acurácia, devem ser comparados os resultados entre o teste pretendido (teste índice) ao teste considerado padrão de referência (padrão ouro). A comparação deve indicar a preditividade do teste índice em prever resultados de modo antecipado, de acordo com a sensibilidade, especificidade e valores preditivos^(8,9)^. Na Fonoaudiologia até o momento, ainda não existia outro protocolo validado para o público de pré-escolares que pudesse ser usado como instrumento de comparação (padrão ouro) na realização de estudos, sendo necessário utilizar uma alternativa padrão-referência.

Segundo o STARD, o padrão-referência é definido como “o melhor método disponível para estabelecer a presença ou ausência da condição alvo” (item 10 –11). Assim, na ausência de instrumentos já padronizados e validados, e populações sem diagnóstico estabelecido, a determinação de “*padrão referência clínico”* pode ser feita a partir da interpretação do olhar de clínicos que atendem a faixa etária, com reconhecida expertise na área^(10)^.

Atualmente existe o Protocolo MMBGR^(2,3)^ voltado para avaliar os aspectos miofuncionais orofaciais na infância, com algumas fases de validação já realizadas. Porém, até o presente momento, não havia sido apresentada a etapa de validade de sua acurácia diagnóstica para pré-escolares (24 a 71 meses de vida), com medidas definidoras de diagnóstico, que viabilizem a tomada de decisões clínicas, prognóstico^(9)^ e encaminhamentos.

Nessa direção, o estudo apresenta a etapa de validação da acurácia diagnóstica referente à faixa etária de pré-escolares do Protocolo MMBGR: Exame Clínico Miofuncional Orofacial com escores e a assertividade do protocolo para encaminhamentos fonoaudiológicos e/ou multidisciplinares.

## MÉTODOS

Estudo do tipo validação, prospectivo e de conveniência, referente à acurácia diagnóstica do Protocolo^(3)^, referente à faixa etária de 24 a 71 meses de idade. Houve anuência das autoras do protocolo original e foi aprovado pelo Comitê de Ética em Pesquisa (CEP) da Universidade Federal de Sergipe, sob número de parecer 5.147.320.

A etapa de validação da acurácia diagnóstica foi possível de ser realizada a partir da proposição anterior do instrumento, ocasião que foram obtidas imagens estáticas e dinâmicas de pré-escolares que frequentavam instituições públicas (creches e hospitais universitários) de dois estados do Brasil, um da região sudeste e outro da região nordeste, mediante autorizações e assinaturas do Termos de Consentimento Livre e Esclarecido (TCLE). Esse banco de imagens já havia sido constituído pelos pré-escolares que atendiam aos critérios de inclusão na pesquisa anterior^(3)^, que era não possuir queixas fonoaudiológicas, diagnósticos neurológicos ou transtornos do desenvolvimento, indicados por responsáveis dos ambientes de coleta.

Foi determinado o tamanho amostral com o objetivo de testar a hipótese de eficácia diagnóstica do método em estudo. O delineamento estatístico buscou detectar uma sensibilidade esperada de 80% (H1), em contraposição a uma hipótese nula de 50% (H0), mantendo-se um poder estatístico de 80% e um nível de significância de 5% (unicaudal). Considerou-se a incerteza quanto à proporção real de indivíduos doentes na população acessada. Assumindo-se uma prevalência estimada de 50% para a condição clínica de interesse, a aplicação dos parâmetros de poder e erro alfa na fórmula de dimensionamento amostral resultou na definição de pelo menos 36 participantes a serem incluídos no estudo. Este quantitativo foi estabelecido para oferecer a probabilidade adequada de rejeição da hipótese nula, caso a performance diagnóstica atinja a magnitude esperada^(11)^.

Para a emissão de pareceres da presente pesquisa, sobre as imagens dos pré-escolares, foram recrutados fonoaudiólogos a partir da análise de seus currículos lattes, conforme os critérios de inclusão da pesquisa, cujo profissional deveria ser especialista em MO e atuar junto à população pré-escolar. Para tanto, realizou-se o levantamento dos profissionais elencados como especialistas no site do Conselho Federal de Fonoaudiologia (CFFa), por diferentes regiões do país; e em seguida houve o contato com cada profissional, por meio de aplicativo de mensagens instantâneas e endereço eletrônico (e-mail). Aqueles que manifestaram interesse receberam, via e-mail, os materiais contendo o TCLE e o *link* para a impressão dele, conforme preconizado pelo CEP.

Ainda por e-mail, foi encaminhado o *link* para o formulário de caracterização profissional, onde a anuência para participação do estudo deveria ser sinalizada, e outro *link* correspondente ao preenchimento da avaliação referente aos casos clínicos do banco de dados a serem analisados. Foi realizado o compartilhamento da nuvem que continha imagens estáticas e dinâmicas dos pré-escolares a serem avaliados.

A distribuição dos vídeos, entre os fonoaudiólogos especialistas, foi feita de modo aleatório respeitando-se a distribuição por faixa etária, logo a quantidade de imagens variou conforme o número de sujeitos pertencentes a cada grupo (G1 ou G2). Foi respeitada a faixa etária que o profissional atuava, mantendo-o sempre com o mesmo grupo etário, G1 - 24 meses a 35 meses e 29 dias (n=36); G2 - 36 meses a 71 meses e 29 dias (n=96).

Os profissionais tiveram autonomia para impor o seu próprio ritmo de avaliações. E os pesquisadores colocaram-se à disposição quanto às eventuais dúvidas, o que poderia ser, inclusive sobre dificuldades dos fonoaudiólogos em realizar as análises no tempo estimado. Se fosse o caso, esse prazo poderia ser expandido.

### Etapas anteriores

O Protocolo MMBGR passou por outras etapas de validação, abrangendo as faixas etárias de lactentes (seis a 23 meses) e pré-escolares (24 a 71 meses): validade de conteúdo do instrumento e validade baseada nos processos de respostas com aplicação do Exame Clínico e realização da confiabilidade do teste^(2,3)^, época na qual foi construído o banco de dados de imagens, conforme as orientações gerais que constam no instrutivo do Protocolo MMBGR lactentes e pré-escolares, seguindo os aspectos do protocolo a serem considerados, de acordo com a faixa etária: com registro estático (JPEG) e dinâmico (MP4)^(2)^, utilizando-se câmera digital (Panasonic Compact-VHS Palmcorder) posicionada em mãos, com imagem aproximada da região orofacial (lente Macro Led Ring Flash HD)^(3)^. Todos os procedimentos descritos, incluindo o recrutamento dos profissionais, seguiram o mesmo formato da pesquisa que envolveu o estudo da acurácia diagnóstica do instrumento para faixa etária de lactentes^(10)^. Ou seja, de acordo com a análise dos dados e, considerando os resultados obtidos, optou-se pela apresentação da acurácia diagnóstica, conforme particularidades relacionadas ao desenvolvimento de cada faixa etária, sendo a população de pré-escolares aqui abordada.

### Constituição dos grupos de participantes (pré-escolares) e domínios avaliados

As imagens (estáticas e dinâmicas) dos pré-escolares foram distribuídas em dois grupos: G1 - 24 meses a 35 meses e 29 dias (n=36); G2 - 36 meses a 71 meses e 29 dias (n=96).

A distribuição dos grupos seguiu a mesma padronização do protocolo original^(3),^ inclusive quanto aos itens e domínios avaliados. Assim, todos os itens analisados eram comuns às duas faixas etárias, exceto aspectos gerais da fonoarticulação (pertencente à fala), cuja proposta original do instrumento é que sejam considerados a partir dos 36 meses de idade. Os autores do instrumento consideram que existe um processo gradual de amadurecimento da fonoarticulação, sendo que com menos de 36 meses há características inerentes ao desenvolvimento que não poderiam ser avaliadas e/ou caracterizadas como Distúrbio Miofuncional Orofacial (DMO), e que aspectos de linguagem também poderiam ser aprofundados em protocolos específicos^(11-14)^.

Quantos aos domínios, o exame extraoral continha itens relacionados à face, aos lábios e a mandíbula; o exame intraoral, direcionado à mucosa dos lábios, bochechas, língua, palato, tonsilas palatinas, dentes e oclusão. Havia ainda a emissão de parecer acerca dos dados relacionados aos tônus de lábios, língua, região do mento e bochechas. Para análises das funções estomatognáticas, foram disponibilizadas as imagens dinâmicas das funções de respiração, mastigação, deglutição de líquido e sólido e fala. Como itens finais do formulário, o avaliador deveria indicar, no domínio geral, se o caso/indivíduo avaliado apresentara ou não DMO, se houve dificuldade para preenchimento do formulário e se havia necessidade de encaminhamentos fonoaudiológicos e/ou multidisciplinares (Odontologia, Otorrinolaringologia, entre outros).

### Acurácia Diagnóstica: teste índice e teste padrão ouro

#### Teste índice

Na etapa anterior referente à validade baseada nos processos de respostas, com a realização da confiabilidade do teste^(2,3)^, fonoaudiólogos calibrados avaliaram as imagens obtidas dos pré-escolares com o uso do Protocolo MMBGR, sendo verificada a concordância entre e intra avaliador^(3)^ de cada item do instrumento. As avaliações (armazenadas no banco de dados do pesquisador) foram consideradas no presente estudo como “teste índice”.

No teste referenciado como índice, foi considerada 100% da amostra para examinar concordância inter-avaliador, e intra avaliador cerca de 20% a 30% desta amostra, de cada faixa etária (39 casos escolhidos aleatoriamente). Essas reavaliações (reteste) feitas pelo mesmo avaliador, ocorreram com o intervalo mínimo de 15 dias da avaliação inicial, evitando que a avaliação anterior influenciasse no desempenho^(3)^. Nesse sentido, utilizou-se a concordância inter e intra examinadores pelo Coeficiente de Correlação Intraclasse CCI, conforme estudo anterior, classificando-se em pobre (inferior a 0,4), de razoável a boa (entre 0,4 e 0,7) e excelente (maior que 0,7)^(3)^.

Na análise da confiabilidade inter avaliador, na faixa etária de 24–35 meses, observou-se concordância pobre para o exame extraoral (CCI=0,26) e para o item tônus (CCI=0,13), enquanto o exame intraoral apresentou concordância excelente (CCI=0,75); os itens respiração, mastigação, deglutição e fala apresentaram concordância razoável a boa, com CCIs de 0,40, 0,56, 0,62 e 0,44, respectivamente. Já na faixa etária de 36–71 meses, o exame extraoral apresentou concordância razoável (CCI=0,52), enquanto o exame intraoral e o item tônus apresentaram concordância pobre (CCI=0,39 e 0,34, respectivamente); observou-se concordância excelente para o item respiração (CCI=0,75) e manutenção de concordância razoável a boa para mastigação, deglutição e fala, com CCIs de 0,45, 0,49 e 0,65, respectivamente^(3).^

Na análise da confiabilidade intra avaliador, na faixa etária de 24–35 meses, o exame extraoral, o exame intraoral e o item fala apresentaram concordância excelente (CCI=0,79; 0,88; 0,88, respectivamente), enquanto os itens tônus (CCI=0,40), respiração (CCI=0,48) e deglutição (CCI=0,51) apresentaram concordância razoável a boa, e mastigação mostrou concordância pobre (CCI=0,38). Já na faixa etária de 36–71 meses, observou-se concordância excelente para os itens exame extraoral (CCI=0,87), tônus (CCI=0,75), respiração (CCI=1,00), deglutição (CCI=0,92) e fala (CCI=0,80), enquanto o exame intraoral (CCI=0,65) e mastigação (CCI=0,71) apresentaram concordância razoável a boa^(3).^

#### Teste padrão ouro

Foram analisadas as mesmas imagens de pré-escolares, armazenadas no banco de dados do pesquisador, por um grupo de fonoaudiólogos especialistas em MO, denominado “comitê de fonoaudiólogos especialistas (avaliadores)”, com experiência clínica no atendimento de pré-escolares e que não podiam ter tido contato prévio e/ou utilizado o Protocolo MMBGR. Os fonoaudiólogos responderam a questões sobre sua caracterização profissional e realizaram as análises das imagens, de modo individual e separadamente, sem o uso do Protocolo MMBGR, sendo essas avaliações consideradas no presente estudo como “teste padrão ouro”.

Foi proposto que cada caso contasse com a avaliação de um trio de especialistas, que responderam separado e individualmente a dois formulários eletrônicos. O primeiro, de “Caracterização Profissional”, continha as questões: faixa etária, região de atuação, titulação, docência, anos de experiência e atuação com pré-escolares; o segundo referia-se ao “Exame clínico miofuncional orofacial”, e incluía as respostas da avaliação dos casos, com base nas imagens compartilhadas; contendo questões sobre os domínios e eventuais alterações. Quantos aos domínios específicos: exame extraoral (face, lábios e mandíbula), exame intraoral (lábios, bochechas, língua, palato, tonsilas palatinas, dentes e oclusão) e tônus (lábios, língua, mento e bochechas). Para análises das funções orofaciais: respiração, mastigação, deglutição - líquido e sólido e semissólidos, e produção de fones/fonemas. Como itens finais do formulário, o avaliador deveria indicar se o caso/indivíduo avaliado apresentava alterações de MO, se necessitava de tratamento fonoaudiológico, e se havia necessidade de encaminhamentos (Otorrinolaringologia e Odontologia). Havia também o questionamento quanto a ocorrência de problemas para preenchimento do formulário.

As respostas obtidas com o “Teste padrão ouro” foram tabuladas e armazenadas em uma plataforma digital, analisadas intragrupo, e esperada a concordância quanto à presença ou à ausência de DMO de pelo menos dois dos três especialistas que analisaram cada caso. No grupo padrão ouro não foram atribuídos escores na avaliação, sendo que o avaliador apenas afirmava de modo dicotômico (sim/não) se havia ou não alteração quanto ao domínio apresentado.

### Análises para validação da acurácia diagnóstica

A metodologia empregada seguiu abordagem similar àquela utilizada para a faixa etária de lactentes^(10)^, comparando os resultados obtidos pelo “teste padrão-ouro” e pelo “teste índice”. O objetivo foi verificar a efetividade do processo avaliativo de pré-escolares por meio do Protocolo de Avaliação Miofuncional Orofacial MMBGR - Lactentes e Pré-escolares (teste índice), quando comparado à avaliação clínica realizada sem o uso do referido protocolo (teste padrão-ouro), bem como determinar sua capacidade preditiva.

Para a validação da acurácia diagnóstica foi essencial a determinação da preditividade. Assim, empregou-se a curva *Receiver Operating Characteristic* (ROC)^(7)^ para a identificação da área sob a curva (AUC) e a definição de pontos de corte sensíveis para alterações nos itens avaliados pelo Protocolo de MMBGR^(3)^.

A partir da identificação de cada ponto de corte, foram calculados os valores de sensibilidade e especificidade. Destaca-se que pode não haver relação entre DMO e os escores do protocolo por domínio, quando a AUC for igual a 0,5, conforme avaliado pelo teste Z para proporções^(15)^. A análise da AUC permite estimar a probabilidade de correta identificação dos indivíduos com alterações (verdadeiros positivos) e daqueles sem alterações (verdadeiros negativos). Valores de AUC próximos de 1,0 indicam melhor desempenho do teste, refletindo elevada sensibilidade e especificidade^(16)^.

A sensibilidade representa a porcentagem de casos corretamente identificados como positivos para alterações, sendo calculada a soma de verdadeiros positivos e falsos negativos. A especificidade, por sua vez, indica a porcentagem de indivíduos corretamente identificados como não portadores da alteração, sendo calculada pela soma de verdadeiros negativos e falsos positivos^(17)^.

O valor preditivo positivo (VPP) corresponde ao percentual de indivíduos efetivamente alterados (verdadeiros positivos) calculado conforme a soma de verdadeiros positivos e falsos positivos. O valor preditivo negativo (VPN) reflete a porcentagem de indivíduos saudáveis corretamente identificados pelo teste como não portadores de alterações (verdadeiros negativos), o calculado conforme a soma de verdadeiros negativos e falsos negativos^(18,19)^.

A análise estatística foi conduzida utilizando o software R Core Team 2022 (Versão 4.2.1). Os resultados são apresentados de forma objetiva, com AUC representando a área sob a curva ROC, IC 95% indicando intervalo de confiança de 95%, SE para sensibilidade, ES para especificidade, ACC para acurácia, além de VP (verdadeiro positivo), FN (falso negativo), FP (falso positivo) e VN (verdadeiro negativo). Ademais, a técnica de Maximização da Sensibilidade com Especificidade Mínima (*Constrained Maximum Sensitivity*) foi utilizada, incluindo os cálculos dos VPP e VPN.

Para a interpretação dos resultados, considerando que um volume reduzido de dados pode comprometer a confiança na classificação, os valores foram adaptados à realidade do estudo. Assim, classificou-se como "não ideal" os valores abaixo de 60%, "razoável" aqueles entre 60% e 80%, e "ideal" aqueles superiores a 80%^(20)^.

## RESULTADOS

Seguem apresentados os resultados das análises realizadas por fonoaudiólogos especialistas, que participaram na condição de avaliadores, distribuídos conforme análise dos dois grupos etários: G1 composto por 36 casos (faixa etária de 24 a 35 meses e 29 dias) e G2 composto por 96 casos (faixa etária de 36 até 71 meses e 29 dias).

Estimava-se a quantidade de 12 fonoaudiólogos para participarem do estudo, distribuídos por grupos de pré-escolares a serem analisados. Entretanto, teve-se que ampliar o número de convites, pois no decorrer do estudo houve a necessidade de ingresso de novos avaliadores com função de complementar ou substituir aqueles que, por motivos extrínsecos/particulares, declinaram a participação antes de finalizarem as análises de todos os casos clínicos. No total, 14 fonoaudiólogos integraram a pesquisa.

Em média, cada especialista analisou de 31 a 40 casos, incluindo imagens estáticas e dinâmicas dos pré-escolares, que compunham o banco de dados da pesquisa. Apenas os especialistas que entraram para completar os grupos, devido à necessidade de substituição de profissional, receberam o drive com um número reduzido de casos, visto que a pesquisa já estava em andamento.

Durante o estudo, foi traçado o perfil socioprofissional dos participantes que compuseram o “comitê de fonoaudiólogos especialistas (avaliadores)” do teste padrão ouro, cujos os dados seguem apresentados na Tabela 1.

**Tabela 1:** Perfil socioprofissional do comitê de fonoaudiólogos especialistas

**Faixa etária**	**% (n)**
31 e 40 anos	28,6 (4)
41 e 50 anos	50,0 (7)
51 e 60 anos	07,1 (1)
61 e 70 anos	14,3 (2)
**Região de exercício profissional**	
Norte	14,3 (2)
Nordeste	28,6 (4)
Centro – oeste	21,4 (3)
Sudeste	28,6 (4)
Sul	07,1 (1)
**Titulação**	
Doutor	50,0 (7)
Mestre	35,7 (5)
Especialista	14,3 (2)
**Docência (tempo de exercício)**	
Não Docente	21,4 (3)
Menos de 05 anos	07,1 (1)
Entre 05 e 10 anos	28,6 (4)
Entre 11 e 15 anos	14,3 (2)
Entre 16 e 20 anos	14,3 (2)
Entre 21 e 25 anos	00,0 (0)
Mais de 25 anos	14,3 (2)
**Tempo de experiência em Fonoaudiologia na motricidade orofacial**	
Entre 11 e 15 anos	28,6 (4)
Entre 16 e 20 anos	14,3 (2)
Entre 21 e 25 anos	28,6 (4)
Mais de 25 anos	28,6 (4)
**Tempo de atuação na área de Motricidade Orofacial com Pré-escolares**	
Entre 05 e 10 anos	07,1 (1)
Entre 11 e 15 anos	35,8 (5)
Entre 16 e 20 anos	21,4 (3)
Entre 21 e 25 anos	07,1 (1)
Mais de 25 anos	28,6 (4)

**Legenda:** % = Percentual; n = número de observações

Foram estabelecidos os seguintes domínios para a análise estatística da MO dos pré-escolares: estruturas orofaciais (extra oral e intra oral), tônus e funções orofaciais (respiração, deglutição, mastigação e fala), com atribuição de escores; além dos domínios quanto a alterações que indicam DMO e necessidade de encaminhamento para tratamento fonoaudiológico e/ou outros encaminhamentos multidisciplinares.

Quanto aos domínios pertencentes à avaliação miofuncional orofacial, seguem na Tabela 2 os valores encontrados a partir da análise dos pareceres emitidos pelo comitê de especialistas, com respectiva representação gráfica da curva ROC na Figura 1.

**Tabela 2:** Apresentação dos valores de acurácia diagnóstica, conforme valores de sensibilidade e especificidade, referente aos domínios da avaliação miofuncional orofacial em pré-escolares

**Domínio**	**Faixa Etária (meses)**	**AUC (IC95%)**	**Valor de Corte**	**Nota máxima**	**SE**	**ES**	**ACC**	**VP**	**FN**	**FP**	**VN**	**VPP**	**VPN**
Estruturas orofaciais	24-35	0,754 (0,499-1,000)	6	76	100	25,0	91,7	32	0	3	1	91,2	50,0
	36-71	0,688 (0,570-0,806)	8	76	96,0	9,1	76,4	71	3	20	2	78,0	40,0
Tônus	24-35	0,570 (0,376-0,764)	1	6	73,9	38,5	61,1	17	6	8	5	68,0	45,0
	36-71	0,700 (0,596-0,805)	1	6	92,2	33,3	64,6	47	4	30	15	61,0	78,9
Funções Orofaciais	24-35	0,697 (0,495-0,899)	7	53	96,5	14,3	80,6	28	1	6	1	82,3	50,0
	36-71	0,774 (0,679-0,869)	9	68	91,4	11,5	69,8	64	6	23	3	73,6	33,3
Motricidade Orofacial (DMO)	24-35	0,590 (0,385-0,795)	15	135	96,1	0	69,4	25	1	10	0	71,4	0
	36-71	0,788 (0,691-0,886)	22	150	97,2	16,7	77,1	70	2	20	4	77,8	66,7

**Legenda:** AUC = Área abaixo da curva ROC; IC95% = Intervalo com 95% de Confiança; SE = Sensibilidade; ES = Especificidade; ACC = Acurácia; VP = Verdadeiro Positivo; FN = Falso Negativo; FP = Falso Positivo; VN = Verdadeiro Negativo. Maximização da Sensibilidade com Especificidade mínima (Constrained Maximum Sensitivity); VPP = Valor Preditivo Positivo; VPN = Valor Preditivo Negativo; DMO = Distúrbio Miofuncional Orofacial

**Figura 1:** Representação gráfica das curvas ROC dos domínios da avaliação miofuncional orofacial em pré-escolares e encaminhamentos, conforme faixa etária (em meses).

Segue o resumo do Exame Clínico do Protocolo de Avaliação Miofuncional MMBGR - Lactentes e Pré-escolares utilizado para registro da avaliação miofuncional orofacial em pré-escolares, com inserção dos respectivos pontos de corte obtidos no presente estudo. (Quadro 1)

**Quadro 1:** Resumo do Exame Miofuncional Orofacial – MMBGR - Lactentes e Pré-escolares, com inserção dos valores de ponto de corte por domínios em pré-escolares, conforme faixa etária

**Faixa etária (meses)**	**24-35 (2 anos)**	**36-71 (3-5 anos)**
**EXAME EXTRAORAL (melhor resultado = 0 e pior = 20)**		
Face (*melhor resultado = 0 e pior = 10)*	[ ]	[ ]
Lábios (*melhor resultado = 0 e pior = 9)*	[ ]	[ ]
Mandíbula (*melhor resultado = 0 e pior =1)*	[ ]	[ ]
**EXAME INTRAORAL (melhor resultado = 0 e pior = 56)**		
Lábios *(melhor resultado = 0 e pior = 5)*	[ ]	[ ]
Bochechas *(melhor resultado = 0 e pior = 8)*	[ ]	[ ]
Língua *(melhor resultado = 0 e pior = 16)*	[ ]	[ ]
Palato *(melhor resultado = 0 e pior = 10)*	[ ]	[ ]
Tonsilas palatinas *(melhor resultado = 0 e pior = 4)*	[ ]	[ ]
Dentes e Oclusão *(melhor resultado = 0 e pior = 13)*	[ ]	[ ]
**RESULTADO EXAME EXTRAORAL + INTRAORAL**	**[ ]**	**[ ]**
**PONTO DE CORTE: EXAME EXTRAORAL + INTRAORAL**	**6**	**8**
**TÔNUS (melhor resultado = 0 e pior = 6)**		
Lábios *(superior + inferior) (melhor resultado = 0 e pior = 2)*	[ ]	[ ]
Mento *(melhor resultado = 0 e pior = 1)*	[ ]	[ ]
Língua *(melhor resultado = 0 e pior = 1)*	[ ]	[ ]
Bochechas *(direita + esquerda) (melhor resultado = 0 e pior = 2)*	[ ]	[ ]
**RESULTADO TÔNUS**	**[ ]**	**[ ]**
**PONTO DE CORTE: TÔNUS**	**1**	**1**
**FUNÇÕES OROFACIAIS (melhor resultado = 0 e pior = 53/68)**		
Respiração *(melhor resultado = 0 e pior = 2)*	[ ]	[ ]
Sucção/Deglutição *(melhor resultado = 0 e pior = 22)*	___	___
Mastigação *(melhor resultado = 0 e pior = 13)*	[ ]	[ ]
Deglutição semissólido/sólido *(melhor resultado = 0 e pior = 17)*	[ ]	[ ]
Deglutição pastoso *(melhor resultado = 0 e pior = 22)*	___	___
Deglutição líquido *(melhor resultado = 0 e pior = 15)*	[ ]	[ ]
**RESULTADO FUNÇÕES OROFACIAIS**	**[ ]**	**[ ]**
**PONTO DE CORTE: FUNÇÕES OROFACIAIS**	**7**	**9**
**ESCORE TOTAL OBTIDO (Exame Clínico Miofuncional Orofacial)**	**[ ]**	**[ ]**
**PONTO DE CORTE: Distúrbio Miofuncional Orofacial (DMO)**	**15**	**22**

Quanto aos domínios pertencentes aos encaminhamentos, seguem na Tabela 3 os valores encontrados a partir da análise dos pareceres emitidos pelo comitê de especialistas.

**Tabela 3:** Apresentação dos valores de acurácia diagnóstica, conforme valores de sensibilidade e especificidade, referente aos domínios de encaminhamentos para pré-escolares

**Domínio**	**Faixa Etária (meses)**	**SE**	**ES**	**ACC**	**VP**	**FN**	**FP**	**VN**	**VPP**	**VPN**
Encaminhamento para fonoaudiologia	24-35	73,1	40,0	63,9	19	7	6	4	76,0	36,4
	36-71	77,3	63,3	72,9	51	15	11	19	82,2	55,9
Outros encaminhamentos multidisciplinares	24-35	83,3	33,3	75,0	25	5	4	2	86,2	28,6
	36-71	84,3	23,1	76,0	70	13	10	3	87,5	18,7

**Legenda:** SE = Sensibilidade; ES = Especificidade; ACC = Acurácia; VP = Verdadeiro Positivo; FN = Falso Negativo; FP = Falso Positivo; VN = Verdadeiro Negativo. Maximização da Sensibilidade com Especificidade mínima (Constrained Maximum Sensitivity). VPP = Valor Preditivo Positivo; VPN = Valor Preditivo Negativo

Com a função de resumir os valores de acurácia diagnóstica e conceito, conforme domínios da avaliação miofuncional orofacial de pré-escolares e encaminhamentos, segue a Tabela 4.

**Tabela 4:** Resumo dos valores de acurácia diagnóstica e conceito, conforme domínios da avaliação miofuncional orofacial de pré-escolares e encaminhamentos, por faixa etária

**Domínio**	**Acurácia/conceito 24 a 35 meses**	**Acurácia/conceito 36 a 71 meses**
Estruturas orofaciais	91,7 - Ideal	76,4 - Razoável
Tônus	61,1 - Razoável	64,6 - Razoável
Funções Orofaciais	80,6 - Ideal	69,8 - Razoável
Motricidade Orofacial (DMO)	69,4 - Razoável	77,1 - Razoável
Encaminhamento para Fonoaudiologia	63,9 - Razoável	72,9 - Razoável
Outros Encaminhamentos (Multidisciplinares)	75,0 – Razoável	76,0 - Razoável

**Legenda:** DMO = Distúrbio Miofuncional Orofacial

## DISCUSSÃO

O Protocolo de Avaliação Miofuncional Orofacial MMBGR – Lactentes e Pré-escolares é um instrumento avaliativo importante na área de MO , com atribuição de escores que permite diagnosticar sujeitos com DMO para a faixa etária de seis a 71 meses de vida. Conforme as especificidades envolvidas, considerou-se apresentar no presente manuscrito os dados pertencentes exclusivamente à população de pré-escolares, incluindo o detalhamento por subgrupos por faixas etárias (Grupo 1: 24 a 35 meses e 29 dias; Grupo 2: 36 a 71 meses e 29 dias).

O perfil dos avaliadores que compuseram o comitê de especialistas foi exclusivamente feminino, com idades a partir de 31 anos. O fato de terem tempo de experiência na área de MO entre 11 e mais de 25 anos, titulação que abrange da especialização em MO até o doutoramento, e experiência que retrata ampla expertise no atendimento clínico de pré-escolares (a maioria de 92,9% com mais de 11 anos de tempo de atuação com essa faixa etária); indica que as imagens foram analisadas por profissionais capacitados e com olhar diferenciado para esse público. A diversidade de locais de atuação do comitê de especialistas atingiu todas as regiões da federação, sendo que na formação dos trios para cada grupo etário, os avaliadores eram de diferentes estados, o que permitiu que a análise do instrumento abrangesse olhares e vivências distintas das variadas regiões do Brasil.

Considera-se que o perfil profissional do comitê de especialistas, que abrangeu vasta experiência docente e clínica na área de MO com pré-escolares, juntamente com a diversidade de locais de atuação, possibilitou que as análises das imagens fossem executadas com o rigor preconizado dos procedimentos desenhados para validação, o que aumenta o valor clínico relacionado ao conteúdo do instrumento e o nível de cientificidade do trabalho^(1)^.

Houve a necessidade de 14 fonoaudiólogos, devido ao quantitativo de participantes da pesquisa; de modo a não ultrapassar o limite de imagens a serem analisadas por cada profissional, optando por um N razoável, sem causar exaustão que comprometesse a pesquisa e/ou bem estar dos profissionais.

No que tange ao domínio de estruturas orofaciais do Protocolo MMBGR, foram obtidos resultados ideal para sensibilidade, sendo capaz de identificar pré-escolares com alteração nessas estruturas, quando alteradas. A especificidade obtida foi não ideal, indicando que o teste é pouco especifico para predizer sujeitos sem alteração na ausência delas. Houve maior evidência em VPP do que em VPN. Isso mostra que o instrumento consegue identificar melhor os pré-escolares quando há alteração. Considera-se que em pré-escolares as alterações nas estruturas orofaciais são mais evidentes do que a ausência delas; sendo que o instrumento consegue identificá-las assertivamente, cumprindo o papel que se propõe para o diagnóstico^(21)^.

A acurácia diagnóstica para estruturas orofaciais foi considerada ideal para o primeiro grupo etário e razoável para pré-escolares acima de 36 meses, evidenciando a capacidade de detectar os sujeitos preditos com alteração em ambos os grupos, com ótima exatidão em pré-escolares de 24 a 35 meses.

Em tônus, foram obtidos níveis de sensibilidade considerados razoável para pré-escolares de 24 a 35 meses e ideal de 35 a 71 meses; sendo o instrumento suficiente na detecção de sujeitos com tônus alterado, quando de fato há alteração. A especificidade não ideal para ambas as faixas etárias, indica dificuldade em identificar pré-escolares sem alterações de tônus. No grupo mais velho os valores de VPP e de VPN evidenciaram que o instrumento consegue identificar de modo razoável tanto a presença como a ausência de alteração de tônus, o que não ocorreu no grupo etário de 24 a 35 meses, cujo VPN foi não ideal, apesar do VPP ter sido razoável. Por outro lado, dado os bons valores de sensibilidade das estruturas orofaciais, considera-se que as suas características poderiam auxiliar para identificar as alterações de tônus, sobretudo em análises feitas por meio de imagens. A ausência de alteração de tônus em sujeitos mais jovens continua a ser um desafio, quando o examinador é privado da palpação direta^(3,22)^.

A acurácia diagnóstica em tônus, manteve-se razoável para todas as faixas etárias, o instrumento teve valores considerados suficientes na exatidão de identificar o que se propôs a avaliar. Ainda assim, considera-se que a avaliação obtida exclusivamente por meio de análise de vídeo pode vir a prejudicar o diagnóstico de alterações do tônus. Na presente pesquisa, as imagens foram compartilhadas, e os avaliadores instruídos a analisarem conforme os critérios que utilizaram para avaliação das demais estruturas dinâmicas. Sendo assim, os avaliadores tiveram liberdade de interpretação conforme sua expertise; e mesmo com a possível limitação, o avaliador fez a apreciação assistindo à imagem dinâmica. Recomenda-se novos estudos com *amostra probabilística* e uso do instrumento para registrar a avaliação em tempo real^(2,3)^.

As funções orofaciais tiveram sensibilidade consideradas ideais para todas as faixas etárias, o instrumento mostrou-se eficaz na identificação de sujeitos com alteração na presença delas. A especificidade foi não ideal, não sendo o instrumento capaz de identificar sujeitos sem alterações na ausência delas. Mais uma vez, houve maior evidência na proporção de VPP do que VPN. A melhor identificação de sujeitos com alteração, o que não ocorreu nos casos não alterados, pode estar relacionada ao próprio amadurecimento fisiológico das funções orofaciais, sendo que os padrões "atípicos" tendem a ser evidentes e com maior chance de serem diagnosticados^(21)^. Por outro lado, a ausência real de alterações não são bem detectadas pelo instrumento, o que coincide com o seu objetivo que é avaliar e diagnosticar o DMO, e não a ausência dele.

Os valores de acurácia das funções orofaciais estiveram razoável para os pré-escolares de 36 a 71 meses, e ideal para àqueles de 24 a 35 meses, evidenciando que o instrumento tem valor no diagnóstico em ambos os grupos dos pré-escolares, com importante papel na clínica da MO, reforçando que a atuação com as funções orofaciais são da expertise do fonoaudiólogo^(23)^.

O domínio referente à alteração de MO diz respeito à presença ou não de DMO. Foram obtidos valores de sensibilidade ideal e especificidade não ideal para todas as faixas etárias de pré-escolares. Assim, o instrumento mostrou-se suficiente para identificar sujeitos com alterações e insuficiente para descartar aqueles sem alterações. O VPP indicou que todos os sujeitos com alterações foram identificados de modo razoável, no entanto houve discrepância nos valores de VPN, quando os pré-escolares de 36 a 71 meses sem alterações puderam ser identificados de maneira razoável.

Os achados quanto ao DMO são condizentes àqueles evidenciados em todos os demais domínios do instrumento, existindo maior capacidade do instrumento predizer sujeitos com alteração do que a ausência dela. Possivelmente, o desenvolvimento do cranio e região orofacial, juntamente com mudanças neurológicas, cognitivas e comportamentais nos primeiros anos de vida, influenciem na identificação dos problemas existentes, os quais tendem a ficar mais evidentes com o avanço da idade^(22)^.

O resultado da acurácia para DMO foi razoável para ambas as faixas etárias, sendo o instrumento capaz de diagnosticar todos os pré-escolares com alterações na presença delas. Reforçando a importância da validação realizada quanto ao Exame Clínico com escores, do Protocolo de Avaliação Miofuncional Orofacial MMBGR Lactentes e Pré-escolares, para a faixa etária de 24 a 71 meses, uma vez que esse material pode auxiliar o clínico a estabelecer diagnóstico em MO de modo padronizado e consistente.

Os valores de escores, referentes aos pontos de corte de cada domínio do Protocolo MMBGR, servem para indicar alterações específicas por domínio^(24)^, culminando no ponto de corte final, indicativo de DMO.

Em relação às estruturas orofaciais, o instrumento permite atribuir escores de 0 (melhor resultado) até 20 (pior resultado) no exame extraoral, e de 0 (melhor resultado) até 56 (pior resultado) para intraoral; e a nota de corte geral ficou estabelecida como 6 para G1 e 8 para G2. Nota-se que valores baixos, como 6 e 8, já sinalizam problemas nas estruturas, o que é importante, considerando a intrínseca relação entre alterações nas estruturas e funções orofaciais^(4)^ e sua influência no diagnóstico do DMO.

Ademais, isso ocorre para tônus que estabelece pontuação entre 0 (melhor resultado) e 6 (pior resultado), e a obtenção de 1 ponto como nota de corte já aponta alteração. É notório que alterações no tônus costumam afetar o funcionamento de todo o sistema estomatognático^(4)^, o que justifica a atribuição do baixo valor de corte no protocolo aqui estudado^(23)^.

Considerando as funções orofaciais, os resultados variam entre 0 (melhor resultado) e 53 (pior resultado) para G1 e entre 0 (melhor resultado) e 68 (pior resultado) para G2, tendo sido estabelecido pontos de corte 7 (G1) e 9 (G2) como indicativo de alteração. Mais uma vez, baixas pontuações já sinalizam ao clínico a necessidade de atenção, e explicitam a íntima relação entre estruturas e funções orofaciais^(4)^.

Quanto aos escores totais para DMO, foram atribuídos os valores de 15 (24 e 35 meses e 29 dias) e de 22 (36 meses e 71 meses e 29 dias) para indicar a presença do distúrbio e a necessidade de acompanhamento fonoaudiológico e/ou multidisciplinares. Valores mais altos de ponto de corte em pré-escolares mais velhos coincide com a possibilidade de atribuir maiores escores em alguns domínios específicos do protocolo quando as idades são mais avançadas.

Ter um protocolo com escores, validado quanto à sua acurácia diagnóstica, com estabelecimento de ponto de corte para DMO é um avanço na área de MO voltada ao público infantil, pois proporciona tanto o diagnóstico como permite vislumbrar sobre necessidade de encaminhamentos fonoaudiológicos e multidisciplinares. O diagnóstico e tratamento para DMO em idades iniciais da vida são relevantes, pois podem contribuir para o pleno desenvolvimento do ser humano nas demais fases da vida^(6)^.

Em suma, o protocolo tem como foco o registro de aspectos inerentes à avaliação oromiofuncional, conforme a análise das estruturas orofaciais (estáticas e móveis), do tônus e das respectivas funções orofaciais, para o estabelecimento de diagnóstico de DMO, com a atribuição de escores. Além disso, também proporciona o raciocínio clínico sobre a necessidade (ou não) de encaminhamentos**.**

A capacidade do instrumento em orientar possíveis encaminhamentos fonoaudiológicos e/ou multidisciplinares foi analisada com base em seus diferentes domínios, ainda que não envolvesse a atribuição de escores específicos.

A necessidade de encaminhamento para Fonoaudiologia foi avaliada devido à sua relevância na reabilitação de indivíduos com possíveis alterações, contribuindo para a definição de intervenções adequadas. Nos dois grupos de pré-escolares a sensibilidade foi classificada como razoável, evidenciando que indivíduos que precisavam de encaminhamento foram identificados e a especificidade considerada não ideal e razoável, apontando que a identificação de sujeitos sem alteração não é tão evidente quando esses têm menor idade, tal como ocorreu no estudo realizado com a faixa etária de lactentes^(10)^.

Houve maior evidência na proporção de VPP do que em VPN, sendo o instrumento capaz de orientar sobre a necessidade de encaminhamentos na presença das alterações. Estima-se que em idades mais avançadas, as possíveis manifestações miofuncionais orofaciais tendem a ser menos toleradas pelo clínico, resultando em uma maior incidência de encaminhamentos^(4)^.

O resultado da acurácia diagnóstica no indicativo de acompanhamento fonoaudiológico para pré-escolares foi razoável, deste modo, o instrumento pode ser reconhecido como um recurso confiável para determinar necessidade de encaminhamentos fonoaudiológicos.

No que se refere à necessidade de encaminhamentos multidisciplinares (Odontologia, Otorrinolaringologia, entre outros) houve sensibilidade ideal e especificidade não ideal, sendo mais evidente a capacidade do instrumento em identificar pré-escolares quando realmente há necessidade do encaminhamento, em ambas as faixas etárias. Os valores de VPP e VPN reiteram a assertividade nos encaminhamentos multidisciplinares de sujeitos quando há presença de alteração. Estes dados evidenciam que o protocolo é capaz de colaborar no direcionamento do caso para outros profissionais, o que pode auxiliar no diagnóstico multidisciplinar e possibilitar que o indivíduo seja visto de modo colaborativo^(25,26)^.

A acurácia razoável do instrumento para encaminhamentos aos demais profissionais pode ser considerada um aspecto positivo. Especialmente na área de Odontologia, essa acurácia pode ter tido relação com a natureza dos itens analisados nos domínios específicos, com prevalência das imagens estáticas que facilitam as análises (dentes e oclusão e frênulo da língua), e na Otorrinolaringologia pela facilidade de visualizar alterações no fluxo aéreo nasal, através do uso do espelho nasal milimetrado^(27,28)^. Por outro lado, na área de Otorrinolaringologia existe uma provável dificuldade de visualização mais detalhada das tonsilas palatinas no exame clínico por parte do fonoaudiólogo, levando à necessidade de encaminhamento para avaliação mais específica.

A falta de encaminhamentos na presença de alterações do sistema estomatognático, pode representar um risco ao desenvolvimento infantil, especialmente em casos cuja abordagem demande o diagnóstico e intervenção a partir da expertise de outras áreas da saúde. Nesse sentido, a capacidade do instrumento de identificar a necessidade de encaminhamento multidisciplinar reforça seu valor tanto para a prática clínica em MO no âmbito fonoaudiológico específico, quanto para o raciocínio interdisciplinar, promovendo uma assistência mais abrangente e integrada.

De modo geral, os achados evidenciam que o Protocolo de Avaliação Miofuncional Orofacial MMBGR – Lactentes e Pré-escolares é inovador para a faixa etária de pré-escolares, pois o estudo de acurácia diagnóstica permitiu definir pontos de corte, complementando as etapas validadas anteriormente. Destacam-se que foram encontrados valores de sensibilidade adequados em todos os domínios do instrumento, demonstrando a capacidade em identificar alterações quando presentes e em direcionar encaminhamentos, tanto fonoaudiológicos quanto multidisciplinares. O fato de apresentar excelente sensibilidade, mas baixa especificidade para estruturas orofaciais — um padrão que se repetiu em outros domínios (tônus, funções, DMO, encaminhamentos) pode eventualmente indicar uma tendência do protocolo a superestimar alterações (maior sensibilidade do que especificidade), o que deve ser interpretado com cautela na prática clínica para evitar encaminhamentos desnecessários.

Por outro lado, os resultados sugerem que o uso do protocolo pode contribuir para detecção precoce e no tratamento oportuno dos DMO, possibilitando melhores desfechos clínicos e estratégias preventivas. Considera-se que seu uso, enquanto instrumento padronizado e validado, deve ser incentivado de modo a complementar processo avaliativo, juntamente com o exame clínico, histórico do paciente e outros instrumentos, se necessário, fortalecendo a tomada de decisão baseada em evidências e evitando diagnósticos errôneos.

A principal dificuldade do estudo foi quanto ao recrutamento dos fonoaudiólogos que estivessem disponíveis a participar do estudo, visto a magnitude de trabalho envolvido nas análises pormenorizadas dos casos. Além disso, a necessidade de substituição de dois fonoaudiólogos para conclusão dos casos, atrasou o cronograma da pesquisa, em relação ao prazo previsto de entrega das análises. Essas limitações foram contornadas com novos convites e anuências, e redistribuição do drive de imagens dos casos ainda não analisados, viabilizando a conclusão da etapa de validação da acurácia diagnóstica. Entretanto, nesse contexto, houve a limitação de reavaliação dos casos pelo mesmo avaliador, e não foi possível realizar a concordância intra-avaliador, como ocorreu anteriormente no teste índice.

Como limitação do estudo, a metodologia de análise dos dados através de imagens gravadas (estáticas e dinâmicas), nem sempre possibilita a melhor visualização e/ou interpretação de determinado domínio avaliado. Recomenda-se que os fonoaudiólogos, ao utilizar o Protocolo MMBGR - Lactentes e Pré-escolares na rotina clínica, façam o registro da situação do exame em tempo real, podendo checar posteriormente as imagens obtidas, tal como preconizado pelos autores do instrumento^(2)^.

Destaca-se que o instrumento demonstrou sensibilidade ideal para a maioria dos domínios em ambas as faixas etárias. A única exceção foi o domínio de tônus na faixa etária de 24 a 35 meses, classificado como razoável. Esse achado sugere a aplicabilidade do instrumento em tempo real, além de destacar a importância do desenvolvimento futuro de estratégias complementares para a avaliação mais precisa desse domínio. A especificidade, predominantemente não ideal, indica que o instrumento apresenta maior eficácia na identificação de sujeitos com alterações quando estas estão presentes. Nesse contexto, destaca-se que o objetivo do Protocolo MMBGR – Lactentes e Pré-escolares é detectar de forma precisa indivíduos com alterações, sugerindo ser uma ferramenta relevante tanto para a prática clínica quanto para pesquisas em MO.

A inexistência de um padrão-referência validado e graduado para DMO é uma limitação estrutural reconhecida na área. De acordo com as recomendações do STARD (Standards for Reporting Diagnostic Accuracy Studies), em situações em que não há um 'padrão ouro' plenamente estabelecido, é adequado empregar o melhor método clínico disponível para determinar a presença ou ausência da condição. No presente estudo, utilizou-se um padrão-referência baseado no consenso de três especialistas em MO, estratégia descrita pelo STARD como forma legítima de adjudicação para reduzir variabilidade inter-observador. O desfecho dicotômico adotado é consistente com a pergunta diagnóstica analisada e com a prática clínica atual. Ainda assim, reconhece-se que a ausência de instrumentos validados de severidade pode influenciar estimativas de acurácia e reforça a necessidade de desenvolvimento futuro de padrões mais estruturados para avaliação da DMO^(29,30)^.

## CONCLUSÃO

O Protocolo de Avaliação Miofuncional Orofacial MMBGR é um instrumento para uso na clínica e na pesquisa fonoaudiológica. Os pontos de corte apresentados possibilitam que o fonoaudiólogo identifique o DMO, utilize os valores como parâmetros para reavaliações e acompanhamento do pré-escolar.

O Protocolo MMBGR apresentou acurácia global razoável para avaliação miofuncional orofacial de pré-escolares, com melhor desempenho para estruturas e funções em crianças de 24–35 meses. O instrumento também se mostrou razoável para subsidiar encaminhamentos fonoaudiológicos e multidisciplinares.

Os resultados sugerem potencial utilidade na clínica da MO, entretanto diante da sua especificidade não ideal quanto a identificação de sujeitos sem DMO, recomenda-se o uso clínico cauteloso, a partir da ampliação de estudos multicêntricos em diferentes populações. Novos estudos de validação transcultural do protocolo, incluindo seus escores são pertinentes.
